# Acute Autonomic Sensory Neuropathy Causing Severe Hyponatremia: A Rare Cause of Syndrome of Inappropriate Antidiuresis

**DOI:** 10.1210/jcemcr/luaf299

**Published:** 2025-12-23

**Authors:** Tatsuo Ogawa, Rieko Kosugi, Akinobu Hori, Masato Kotani, Tatsuhide Inoue, Hiroyuki Ariyasu

**Affiliations:** Center for Diabetes, Endocrinology and Metabolism, Shizuoka General Hospital, Shizuoka 420-8527, Japan; Center for Diabetes, Endocrinology and Metabolism, Shizuoka General Hospital, Shizuoka 420-8527, Japan; Department of Neurology, Graduate School of Medicine and Faculty of Medicine, Kyoto University, Kyoto 606-8507, Japan; Center for Diabetes, Endocrinology and Metabolism, Shizuoka General Hospital, Shizuoka 420-8527, Japan; Center for Diabetes, Endocrinology and Metabolism, Shizuoka General Hospital, Shizuoka 420-8527, Japan; Center for Diabetes, Endocrinology and Metabolism, Shizuoka General Hospital, Shizuoka 420-8527, Japan

**Keywords:** acute autonomic sensory neuropathy, hyponatremia, syndrome of inappropriate antidiuresis

## Abstract

A 25-year-old woman had experienced dizziness and numbness following a recent fever accompanied by upper respiratory symptoms. She was subsequently admitted to our hospital because of impaired consciousness and generalized tonic-clonic seizures. Severe hyponatremia and increased urinary sodium excretion were noted, leading to a diagnosis of syndrome of inappropriate antidiuresis (SIAD), being suspected Guillain-Barré syndrome (GBS) as the cause. However, she did not present with muscle weakness or deep sensory impairment, whereas orthostatic hypotension and the results of 123-I-meta-iodobenzylguanidine scintigraphy suggested the presence of autonomic dysfunction. We diagnosed her with acute autonomic and sensory neuropathy (AASN) rather than GBS. AASN is a neurological disorder closely related to GBS, characterized by an acute onset of sensory and autonomic dysfunction without motor impairment. Because AASN may rapidly progress to a severe form, it is important to recognize that AASN is a contributing cause of SIAD and to manage it appropriately at an early stage.

## Introduction

Syndrome of inappropriate antidiuresis (SIAD), caused by excess antidiuretic hormone (ADH) despite low serum osmolality, is the most common cause of hypotonic hyponatremia. The etiologies of SIAD are diverse. Data from a hyponatremia registry reported the following distribution: malignancies (23.6%), pharmacological agents (17.8%), pulmonary disorders (10.7%), and central nervous system diseases (8.5%) [1]. Among neurological disorders, inflammatory demyelinating diseases, such as Guillain-Barré syndrome (GBS), have been recognized as potential causes of SIAD [2].

Hyponatremia is biochemically classified according to serum sodium concentration as mild (130-135 mmol/L), moderate (125-129 mmol/L), and profound (<125 mmol/L) [3]. Another categorization is acute or chronic hyponatremia; “acute” is defined as duration less than 48 hours, whereas “chronic” is defined as duration greater than 48 hours [4]. “Severe” hyponatremia is defined by the presence of symptoms of cerebral edema such as vomiting, seizures, and impaired consciousness; it warrants emergency treatment [3].

Acute autonomic and sensory neuropathy (AASN) is a rare inflammatory demyelinating disorder of the peripheral nervous system that shares pathophysiological features with GBS [5]. SIAD has occasionally been reported in association with AASN. Like GBS, it is thought to result from an autoimmune response triggered by preceding infection [6]. However, while GBS mainly presents with motor impairment and mild autonomic involvement, AASN is characterized by prominent autonomic and sensory dysfunction with little or no motor weakness [6, 7]. The underlying mechanism of SIAD in AASN remains unclear but may involve autonomic dysfunction between baroreceptors and the hypothalamus [8], as well as concomitant involvement of the central nervous system [7, 9]. The disease typically progresses rapidly within a few days after infection, whereas GBS develops gradually over 1 to 3 weeks [6, 7].

Because autonomic failure or hyponatremia can be life-threatening, early recognition and prompt management are essential. However, AASN remains underrecognized despite its rapid progression and potential severity. Here, we report a rare case of SIAD associated with AASN, which was diagnosed after the onset of hyponatremia, and review the relevant literature.

## Case Presentation

The patient is a 25-year-old female with a height of 151 cm, a weight of 52 kg, and a body mass index of 22.8 kg/m². The patient had no prior psychiatric disorders and was not taking antidepressants, antipsychotics, antiepileptics, or diuretics. She reported no alcohol consumption. On the fifth day before admission, she presented with a fever of 38.6 °C, loss of appetite, dizziness, and numbness in the limbs. On the second day before admission, she presented to our emergency department with leg cramps. Although her serum sodium level was 134 mEq/L (SI: 134 mmol/L) (reference range: 138-145 mEq/L [SI: 138-145 mmol/L]), which is mildly decreased relative to the institutional reference range, she was advised to restrict excessive fluid intake and was discharged home. On the day of admission, she suddenly developed impaired consciousness accompanied by generalized tonic-clonic seizures and was transported to our hospital by ambulance. Her body temperature, blood pressure, and heart rate were 36.2 °C, 126/99 mm Hg, and 74 beats per minute, respectively. Glasgow Coma Scale score was E4V1M5. Neurological examination revealed no nuchal rigidity and no evident cranial nerve abnormalities. Deep tendon reflexes and muscle strength were normal. Regarding superficial sensation, there was numbness in the distal extremities and abnormal perception of the back. On the other hand, deep sensations, such as joint position and vibration, were normal. Serum sodium concentration was 119 mEq/L (SI: 119 mmol/L). A computed tomography scan of the head revealed no abnormalities, and severe hyponatremia was thought to be the cause of the seizures.

## Diagnostic Assessment

Blood and urine tests revealed a hypotonic hyponatremia (serum osmolality <275 mOsm/kg H_2_O [SI: <275 mmol/kg]), inappropriately elevated urine osmolality (>100 mOsm/kg H_2_O [SI: >100 mmol/kg]), urine sodium >30 mEq/L (SI: >30 mmol/L), and normal renal function (Table 1). On physical examination, there was no edema and no clinical signs of dehydration, such as dry mucous membranes or decreased skin turgor, despite orthostatic hypotension attributable to autonomic dysfunction. There were no clinical findings suggestive of adrenal insufficiency or hypothyroidism, and both serum cortisol and thyroid function tests did not indicate deficiencies in these hormones (Table 1). Although measurement of ADH is not required for diagnosing SIAD, we report its value in Table 1 for completeness. Based on these findings, she was diagnosed with SIAD.

**Table 1.** Laboratory data on admission

Parameters	Values (Conventional; SI)	Reference range (Conventional; SI)
**Hematology**		
WBC	8100/µL	3300-18,600/µL
Hb	**12.2 g/dL (122 g/L)**	13.7-16.8 g/dL (138-168 g/L)
Ht	44.2%	40.7-50.1%
Plt	26.5 × 10^4^/µL	15.8-34.8 × 10^4^/µL
**Biochemistry**		
TP	7.9 g/dL (79 g/L)	6.6-8.1 g/dL (66-81 g/L)
Alb	4.1 g/dL (41 g/L)	4.1-5.1 g/dL (41-51 g/L)
AST	**53 IU/L**	13-30 IU/L
ALT	**56 IU/L**	10-42 IU/L
LDH	**227 IU/L**	124-222 IU/L
CK	**176 IU/L**	41-153 IU/L
BUN	**4.0 mg/dL (1.4 mmol/L)**	8-20 mg/dL (2.86-7.14 mmol/L)
Cre	**0.33 mg/dL (29.2 µmol/L)**	0.46-0.79 mg/dL (40.7-69.8 µmol/L)
UA	**1.7 mg/dL (101 µmol/L)**	3.7-7.0 mg/dL (220-416 µmol/L)
Na	**119 mEq/L (119 mmol/L)**	138-145 mEq/L (138-145 mmol/L)
K	3.8 mEq/L (3.8 mmol/L)	3.6-4.8 mEq/L (3.6-4.8 mmol/L)
Cl	**82 mEq/L (82 mmol/L)**	101-108 mEq/L (101-108 mmol/L)
CRP	0.07 mg/dL (700 µg/L)	≦0.14 mg/dL (≦1400 µg/L)
P-Osm	**238 mOsm/kg H₂O (238 mmol/kg)**	275-290 mOsm/kg H₂O（275-290 mmol/kg）
**Endocrinology**		
LH	**6.84 mIU/mL (6.84 IU/L)**	0.79-5.72 mIU/mL (0.79-5.72 IU/L)
FSH	4.11 mIU/mL (4.11 IU/L)	2.00-8.30 mIU/mL (2.00-8.30 IU/L)
E2	20 pg/mL (73.4 pmol/L)	
GH	0.52 ng/mL (0.52 µg/L)	0.13-9.88 ng/mL (0.13-9.88 µg/L)
IGF-I	237 ng/mL (31.0 nmol/L)	109-284 ng/mL (14.3-37.2 nmol/L)
PRL	**35.7 ng/mL (35.7 µg/L)**	4.9-29.3 ng/mL (4.9-29.3 µg/L)
ACTH	**91.5 pg/mL (20.3 pmol/L)**	7.2-63.3 pg/mL (1.6-14.0 pmol/L)
Cortisol	**35.2 µg/dL (971.1 nmol/L)**	7.1-19.6 µg/dL (195.9-540.8 nmol/L)
TSH	**0.23 µIU/mL (0.23 mIU/L)**	0.5-5.0 µIU/mL (0.5-5.0 mIU/L)
Free T3	**2.1 pg/mL (3.23 pmol/L)**	2.3-4.0 pg/mL (3.53-6.14 pmol/L)
Free T4	1.53 ng/dL (19.7 pmol/L)	0.9-1.7 ng/dL (11.6-21.9 pmol/L)
ADH	**4.7 pg/mL (4.34 pmol/L)**	<2.8 pg/mL (<2.58 pmol/L)
PRA	**<0.2 ng/mL/h (<0.15 nmol/L/h)**	0.2-2.3 ng/mL/h (0.15-1.77 nmol/L/h)
PAC	**<4.0 pg/mL (<11.1 pmol/L)**	4.0-82.1 pg/mL (11.1-227.8 pmol/L)
Metanephrine	26 pg/mL (131.8 pmol/L)	≦130 pg/mL (≦659.1 pmol/L)
Normetanephrine	37 pg/mL (202.0 pmol/L)	≦506 pg/mL (≦2762 pmol/L)
**Urinalysis**		
U-Na	218 mEq/L (218 mmol/L)	
U-K	34.8 mEq/L (34.8 mmol/L)	
U-Cl	201 mEq/L (201 mmol/L)	
U-Osm	631 mOsm/kg H₂O (631 mmol/kg)	

Abnormal values are shown in boldface. Values are shown as conventional, (SI).

Abbreviations: ADH, antidiuretic hormone; E2, estradiol; PAC, plasma aldosterone concentration; PRA, plasma renin activity; PRL, prolactin.

Magnetic resonance imaging of the head and computed tomography scans of the chest revealed no abnormalities that could cause SIAD. She had no history of taking medications known to induce SIAD. Although no motor deficits were observed at the initial presentation, antecedent events and the neurological symptoms, including numbness in the extremities, were presented. Given that motor dysfunction might emerge later in the clinical course, acute motor and sensory axonal neuropathy, a subtype of GBS, was suspected as the underlying cause of SIAD.

**Figure 1.**
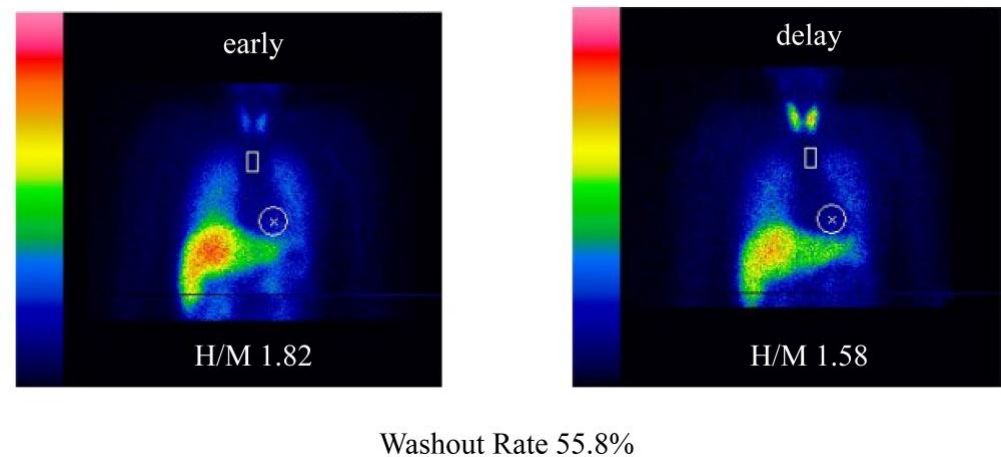
123-I-meta-iodobenzyguanidine (MIBG) myocardial scintigraphy performed on day 48. The image shows decreased cardiac uptake with a heart-to-mediastinum (H/M) ratio of 1.82 (early value) and 1.58 (delayed value), along with an increased washout rate.

A cerebrospinal fluid examination conducted to confirm the diagnosis of acute motor and sensory axonal neuropathy revealed albuminocytologic dissociation. In the present case, anti-ganglioside antibodies, which are reported to be positive in approximately 60% of patients with GBS, were not detected [10]. On the 14th day of hospitalization, neurological examination revealed decreased deep tendon reflexes, whereas no muscle weakness was observed in the extremities. In contrast, the coefficient of variation of the R-R interval was decreased, and the Schellong test demonstrated orthostatic hypotension, indicating that the autonomic nervous dysfunction was predominant (Table 2). Furthermore, 123-I-meta-iodobenzylguanidine scintigraphy demonstrated reduced uptake in both early and delayed phases, along with an increased washout rate. These findings are consistent with autonomic dysfunction (Fig. 1). Although several supportive findings for the diagnosis of GBS were present, the absence of the essential criteria [11], including progressive and symmetrical limb weakness and diminished deep tendon reflexes, made the diagnosis of GBS unlikely. Given the rapid onset of widespread autonomic and sensory neuropathy within 1 day following an antecedent event, and in the absence of any underlying condition suggestive of secondary neuropathy, she was diagnosed with AASN, related diseases of GBS. We concluded that AASN was the underlying cause of SIAD observed in this case.

**Table 2.** Neurological examination

Cerebrospinal fluid	Values	Reference range
Cell count	2/µL (2 × 10^6^/L)	0-5/µL (0-5 × 10^6^/L)
Protein	**45.3 mg/dL (0.45 g/L)**	10-35 mg/dL (0.1-0.35 g/L)
Glucose	70 mg/dL (3.89 mmol/L)	50-80 mg/dL (2.77-4.45 mmol/L)
ADA	<2.0 U/L	
Alb	232 mg/L (0.232 g/L)	
IgG	7.5 mg/dL (0.075 g/L)	
OCB	Not detected	
**CVRR**		
Normal breathing	3.00%	
After deep breathing	**2.12%**	
**The Schellong test**		
At rest	126/87 mm Hg	
Right after waking up	**102/52 mm Hg**	

Abnormal values are shown in boldface. Abbreviations: ADA, adenosine deaminase; CVRR, coefficient of variation of R-R interval; OCB, oligoclonal IgG bands.

## Treatment

At admission, the patient's serum sodium level was less than 125 mEq/L (SI: 125 mmol/L), accompanied by impaired consciousness and seizures. She was diagnosed with severe hyponatremia. The hyponatremia in this case was estimated to have developed within 48 hours, suggesting an acute form.

In acute and severe symptomatic hyponatremia, although vigilance for cerebral edema is required, relatively rapid correction is permissible, and initial treatment with hypertonic saline is indicated. Under close monitoring, the initial target is to increase the serum sodium level by 4 to 6 mmol/L. A strategy for prompt management of unintended overcorrection is essential. In contrast, for chronic hyponatremia, gradual correction at a rate of approximately 5 to 8 mmol/L per day is recommended to minimize the risk of osmotic demyelination syndrome [4].

**Figure 2.**
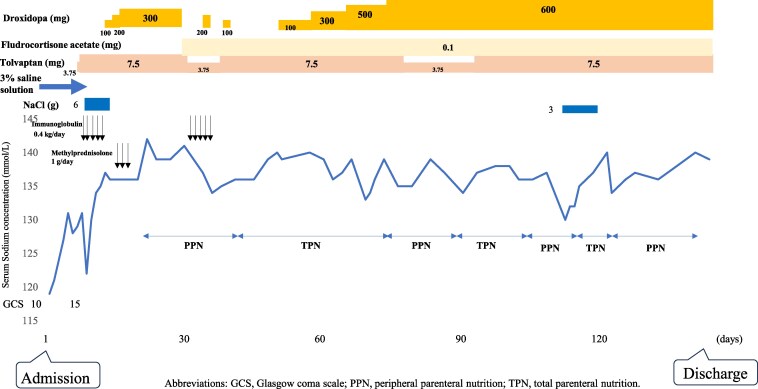
The clinical course of treatment, serum sodium concentration.

As shown in Fig. 2, 3% hypertonic saline was administered to correct the serum sodium level with careful monitoring (a 100-mL infusion was administered twice). The serum sodium level increased by 6 mmol/L within the first 24 hours, accompanied by improvement in the patient's level of consciousness. However, despite fluid restriction, serum sodium levels declined promptly after discontinuation of hypertonic saline. Therefore, oral tolvaptan therapy was initiated on the sixth day of hospitalization and her serum sodium level remained within the range of 130 to 140 mEq/L (SI: 130-140 mmol/L). Consequently, fluid restriction was discontinued, and no further episodes of impaired consciousness or seizures were observed.

Although she was treated with intravenous immunoglobulin and methylprednisolone, there was no significant improvement in sensory disturbances, autonomic dysfunction, and gastrointestinal dysfunction. Approximately 4 months after the start of treatment, she became able to tolerate oral intake, and orthostatic hypotension also improved with droxidopa and fludrocortisone.

## Outcome and Follow-up

Tolvaptan was gradually tapered due to the sustained stability of serum sodium levels and was discontinued 1 year later. Symptomatic treatment for AASN was continued, resulting in gradual improvement of autonomic dysfunction. However, sensory disturbances have persisted.

## Discussion

A literature search using the PubMed search engine identified 5 cases of AASN [6, 9, 12], including the present case, in which AASN was reported in association with SIAD (Table 3). The majority of patients were young women, and the interval between the antecedent event and the onset of neurological symptoms was consistent with that described in the literature. However, in the present case, this interval was notably short, with neurological symptoms developing within a single day. Among the 5 cases with available clinical data, SIAD was transient in 3 cases. Of the remaining 2 cases, 1 could not be followed because of the patient's death, and the other lacked detailed information on the clinical course of SIAD. Although the severity and presentation varied among cases, autonomic dysfunction generally showed a tendency toward improvement.

**Table 3.** Published cases of AASN with SIAD

First author. Year	Study type	Sex	Age (y)	Antecedent events	Duration*^a^*	Serum Na (138-145 mEq/L)[138-145 mmol/L]	CSF protein (10-35 mg/dL)[0.1-0.35 g/L]	Course of SIAD
Koike, H 2010 [6]	Case with review	F	27	URI	2 days	NA	121 mg/dL [1.21 g/L]	NA*^b^*
Koike, H 2010 [6]	Case with review	F	22	Fever	5 days	NA	455 mg/dL [4.55 g/L]	NA*^c^*
Irioka, T 2001 [9]	Case Report	M	57	eruption	7 days	124 mEq/L [124 mmol/L]	159 mg/dL [1.59 g/L]	Transient
Adachi, H 1998 [12]	Case Report	F	26	URI	2 days	124 mEq/L [124 mmol/L]	121 mg/dL [1.21 g/L]	Transient
Present case		F	25	Fever	1 day	119 mEq/L [119 mmol/L]	45.3 mg/dL [0.45 g/L]	Transient

Serum Na levels and CSF protein levels are shown as conventional and [SI].

Abbreviations: CSF, cerebrospinal fluid; NA, not available; URI, upper respiratory infection.

^*a*^From antecedent events to initial symptom.

^*b*^Died of sepsis.

^*c*^Autonomic nervous system disorders have improved.

The pathophysiological mechanism underlying the association between AASN and SIAD remains unclear. In malignancy, paraneoplastic SIAD is primarily mediated by the ectopic secretion of ADH from tumor cells [4] and tends to be persistent. In contrast, in AASN- or GBS-associated SIAD, autonomic afferent dysfunction and potential hypothalamic dysregulation may stimulate ADH release from the hypothalamus. In GBS, several mechanisms have been proposed, including a lowered threshold of hypothalamic osmoreceptors [13], increased renal tubular sensitivity to ADH [14], and afferent autonomic dysfunction of the cardiovascular system involving baroreceptors and volume receptors [8]. In the present case, the presence of autonomic dysfunction and elevated cerebrospinal fluid protein levels suggests that SIAD may have developed through similar mechanisms.

Taken together, these findings suggest that dysfunction of the autonomic and hypothalamic pathways may play a key role in the development of SIAD in AASN. Further evidence supporting central nervous system involvement has been reported in pathological and immunological studies, as described later. Autopsy studies in patients with acute pandysautonomia accompanied by sensory neuropathy have revealed mild chronic meningoencephalitis characterized by perivascular lymphocytic infiltration and microglial nodules in the hippocampal region [15]. In addition, in idiopathic polyneuropathy, hypothalamic damage during the disease course may lead to leakage of ADH into the systemic circulation [8]. Moreover, IL-6 has been reported to stimulate the secretion of ADH from the supraoptic and paraventricular nuclei [16].

This was the only case in which tolvaptan was used to treat SIAD. Tolvaptan is generally recommended as a second-line therapy for SIAD when fluid restriction is ineffective, as endorsed by both the European and US expert guidelines [3, 17], but it was used because hyponatremia persisted even after 3% hypertonic saline infusion. In previous reports, SIAD typically resolved within several days to 1 month. However, in the present case, prolonged autonomic and sensory dysfunction, along with insufficient oral intake likely due to impaired gastrointestinal motility, contributed to recurrent hyponatremia, making it difficult to discontinue tolvaptan.

## Learning Points

- In some cases of AASN, SIAD may occur concurrently. This condition is more common in young women and typically develops acutely within a few days of a preceding infection.
- SIAD associated with AASN can cause acute and severe hyponatremia. Because autonomic dysfunction and hyponatremia can be life-threatening, early recognition and prompt intervention are essential.
- The mechanisms underlying SIAD onset are thought to involve multiple factors, including dysfunction of the autonomic nervous system and the hypothalamus.

## Contributors

All authors made individual contributions to the authorship. A.H. and T.O. were involved in the diagnosis and management of this patient. R.K., M.K., T.I., and H.A. managed the patient. T.O. and H.A. prepared the manuscript. All authors reviewed and approved the final draft of the manuscript.

## Data Availability

Original data generated and analyzed during this study are included in this published article.
